# 534 Allogeneic Cellularized Living Tissue in Pediatric Deep Partial Thickness Burns Reduces Need for Donor Sites

**DOI:** 10.1093/jbcr/irac012.163

**Published:** 2022-03-23

**Authors:** Angela Gibson, Tracee Short, Victor C Joe

**Affiliations:** University of Wisconsin School of Medicine and Public Health, Madison, Wisconsin; Baton Rouge General, Baton Rouge, Louisiana; UCI Health Regional Burn Center, Orange, California

## Abstract

**Introduction:**

Healing potential of deep partial thickness burns is not easily determined early after injury. Prolonged and continued inflammation can contribute to burn wound conversion. Early excision compared to a wait and see approach to wound healing decreases inflammation, shortens length of stay, and decreases painful wound procedures. However, early excision is at the expense of removing tissue that may otherwise heal without autografting. Use of a cryopreserved, allogeneic cellularized scaffold living tissue product (CTP) in pediatric deep partial thickness wounds with dermal elements remaining after excision is an alternative approach to wound closure. CTP may allow early excision without compromising healing potential, thereby minimize donor site morbidity, infectious complications and decrease length of stay. This case series describes the expanded access experience of a CTP on pediatric patients with thermal injury.

**Methods:**

Expanded use approval of a CTP was obtained through the FDA for pediatric patients with burns at three burn centers. Patients were taken to the operating room for excision and grafting of burns at the discretion of the attending surgeon. In wounds with dermal elements present after excision, CTP was grafted to the wound bed (Table 1). Postoperative wound care and follow up was per institutional standards of care.

**Results:**

Six pediatric patients (9 weeks – 13 years old) with mixed depth burns having dermal elements remaining after excision were treated successfully with a CTP. Donor site surface area was reduced in all patients. No serious adverse events related to the CTP were noted in any of the cases. Average time to heal wounds treated with the CTP was approximately 2-3 weeks. In wounds ultimately deemed to be full thickness, CTP was not detrimental to subsequent autograft take. See Table 1.

**Conclusions:**

This case series demonstrates that use of a CTP in pediatric patients is donor site sparing without any serious adverse events. Furthermore, use in full thickness burns is not detrimental to subsequent autograft take. The degree of dermal elements necessary to support wound healing is unknown and requires further study. This case series supports the application for a clinical trial investigating the safety and efficacy of CTP as an adjunct to wound healing in pediatric deep partial thickness burns.

